# Impact of Allergic Diseases or Obstructive Sleep Apnea Risk on Severe *Mycoplasma pneumoniae* Pneumonia in Children: A Clinical Study and Nomogram Construction

**DOI:** 10.3390/jcm15083159

**Published:** 2026-04-21

**Authors:** Zonglang Yu, Jingrong Song, Yu Fu, Rui Li, Ruimeng Ma, Tienan Feng, Mengting Zhang, Shuping Jin, Xiaoying Zhang

**Affiliations:** 1 Shanghai Ninth People’s Hospital, Shanghai Jiao Tong University School of Medicine, No. 639, Zhi Zao Ju Road, Shanghai 200011, China; yuzonglang@sjtu.edu.cn (Z.Y.); songyang92006@163.com (J.S.); fuyu18856030890@163.com (Y.F.); 72300320004@shsmu.edu.cn (R.L.); sakura666@sjtu.edu.cn (R.M.); zmtaaaaa@163.com (M.Z.); 330483200108230066@sjtu.edu.cn (S.J.); 2 Institute of Clinical Medicine, Shanghai Jiao Tong University School of Medicine, No. 227 Chongqing South Road, Shanghai 200025, China; tienan_feng@126.com

**Keywords:** severe *Mycoplasma pneumoniae* pneumonia, allergic diseases, obstructive sleep apnea, RQLQ, PSQ

## Abstract

**Background/Objectives**: This study aimed to investigate the impact of allergic diseases (AD) or obstructive sleep apnea (OSA) risk, as a host factor, on the development of severe *Mycoplasma pneumoniae* Pneumonia (SMPP) in children by analyzing the clinical data of pediatric patients with *Mycoplasma pneumoniae* Pneumonia (MPP). **Methods**: This retrospective study enrolled children hospitalized with *Mycoplasma pneumoniae* pneumonia (MPP) at Shanghai Ninth People’s Hospital from November 2024 to November 2025. Patients were classified into severe (SMPP) and mild (MMPP) groups. Demographic, clinical, laboratory, and questionnaire data were collected and compared between groups. Univariate and multivariate logistic regression analyses were performed to identify independent predictors of SMPP and construct a nomogram. The model was validated for discrimination, calibration, and clinical utility using ROC curves, calibration plots, and decision curve analysis, with internal validation by bootstrap resampling. **Results**: Among the 150 enrolled children with MPP, 35 (23.3%) were classified as severe (SMPP) and 115 (76.7%) as mild (MMPP). Patients with SMPP exhibited significantly higher frequencies of allergic diseases, prolonged fever and steroid use, elevated inflammatory markers (CRP, LDH, D-dimer, ferritin, ALT), and higher PSQ and RQLQ scores (all *p* < 0.05). Disease severity was positively correlated with these clinical, laboratory, and questionnaire-based parameters. Multivariate logistic regression identified allergic diseases, PSQ score, LDH, and ferritin as independent predictors of SMPP. A nomogram incorporating these four factors demonstrated good predictive performance, with an internally validated C-index of 0.827, satisfactory calibration (Hosmer–Lemeshow *p* = 0.116), and clinical utility within a 0–25% threshold probability range on decision curve analysis. **Conclusions**: Children with MPP and comorbid AD or OSA risk are more likely to develop SMPP. Among children aged 6–12 years, RQLQ score is positively correlated with the severity of MPP. AD, PSQ score, LDH, and ferritin are independent risk factors for SMPP. Clinicians should be alert to the development of SMPP when children with MPP present with a history of AD, PSQ score >3.5, LDH >327.50 U/L, or ferritin >120.05 ng/mL. The visual nomogram model constructed by combining these risk factors demonstrates improved predictive performance for SMPP, with high predictive efficacy and accuracy. It has great clinical value and can be used for individualized risk assessment and early intervention. However, our proposed nomogram requires external validation prior to broader implementation.

## 1. Introduction

*Mycoplasma pneumoniae* (MP) is a unique pathogen and ranks as the third most common cause of lower respiratory tract infections worldwide. Respiratory tract infections caused by MP typically exhibit epidemic peaks every 3–7 years, with each outbreak lasting 1–2 years [1,2,3]. *Mycoplasma pneumoniae* pneumonia (MPP) is the leading cause of community-acquired pneumonia (CAP) among children aged ≥5 years in China, although it can also occur in younger children. While MP infection was traditionally considered self-limiting, an increasing number of cases progressing to Severe MPP (SMPP) with significant pulmonary and extrapulmonary complications have been reported in recent years [4].

Most scholars currently believe that direct injury induced by MP and abnormal host immune responses constitute the main pathogenic mechanisms of MP infection. The influence of host factors on the severity of MP infection has received increasing attention. In recent years, the prevalence of allergic diseases (AD) among Chinese children has increased annually. Among these, allergic rhinitis (AR) is a common non-infectious chronic inflammatory disease of the nasal mucosa in children, which is closely related to immune imbalance after exposure to allergens [5]. Obstructive sleep apnea (OSA), a serious sleep-disordered breathing condition in children, has attracted considerable attention from parents and society due to its high prevalence and severe long-term complications [6]. Studies have indicated that patients with OSA are more susceptible to CAP, and patients with severe OSA are more likely to develop severe pneumonia [7]. However, systematic investigations into the association between AD and SMPP, as well as OSA and SMPP, in children remain limited. This study aims to investigate the impact of AD or OSA risk on the occurrence of SMPP in children, so as to facilitate the early clinical identification of high-risk children, improve prognosis, and enhance the quality of life of affected children.

## 2. Materials and Methods

### 2.1. Patient Population

A total of 150 children diagnosed with *Mycoplasma pneumoniae* pneumonia (MPP) at Shanghai Ninth People’s Hospital from November 2024 to November 2025 were enrolled in this study. Written informed consent was obtained from all participants’ guardians. This study was approved by the Ethics Committee of Shanghai Ninth People’s Hospital (Ethics No.: SH9H-2024-T331-1). This study also followed the Strengthening the Reporting of Observational Studies in Epidemiology (STROBE) guideline for observational research. Given that we developed a predictive nomogram, we also adhered to the transparent reporting principles of the Transparent Reporting of a Multivariable Prediction Model for Individual Prognosis or Diagnosis (TRIPOD) statement.

Inclusion Criteria: (1) Diagnosis of MPP confirmed according to the diagnostic criteria outlined in the Guidelines for the diagnosis and treatment of *Mycoplasma pneumoniae* pneumonia in children (2025 edition) [4] released by the National Health Commission. In addition to compatible clinical manifestations and imaging findings, pediatric patients were required to have positive results from at least one of the following laboratory tests: (1). MP-DNA or MP-RNA by nucleic acid amplification test. (2). A single serum MP antibody titer ≥1:160, or a four-fold or greater increase in MP antibody titers in paired serum samples during the illness period, as determined by particle agglutination test. (3). Age between 3 and 14 years, inclusive.

Exclusion Criteria: (1) Evidence of concurrent infection with other bacteria, viruses, or fungi. (2) Comorbid other respiratory conditions (e.g., bronchial foreign body, pulmonary tuberculosis, bronchopulmonary dysplasia, airway malformations), autoimmune diseases, tumors, immunodeficiency disorders, or severe cardiac, hepatic, or renal diseases. (3) Long-term use of corticosteroids or immunomodulators. (4) Incomplete clinical data.

### 2.2. Diagnostic Criteria

Criteria for defining SMPP: SMPP was diagnosed according to the classification criteria outlined in the Guidelines for the diagnosis and treatment of *Mycoplasma pneumoniae* pneumonia in children (2025 edition) [4]. Children with MPP who did not meet the diagnostic criteria for SMPP were classified as having mild MPP (MMPP).

A history of AD was defined as meeting any of the following criteria: (1) previous clinical diagnosis of allergic rhinitis (AR), atopic dermatitis, atopic asthma, or eczema by a specialist physician; (2) confirmed history of inhalation or food allergy.

Criteria for defining AR: AR was diagnosed according to the Guideline for diagnosis and treatment of pediatric allergic rhinitis (2022, revision) [5]. The diagnosis required meeting both of the following criteria: (1) presence of at least two of the following local symptoms: nasal congestion, rhinorrhea, nasal itching, or paroxysmal sneezing, with symptoms persisting or cumulatively lasting for more than one hour daily; (2) positive result from any one of the allergen tests.

Pediatric Rhinoconjunctivitis Quality of Life Questionnaire (PRQLQ): This questionnaire is widely used to evaluate the quality of life in children aged 6–12 years with AR. The authorized Chinese version of the RQLQ is recommended for clinical use. The PRQLQ comprises 23 items across five domains: nose symptoms, eye symptoms, other symptoms, behavioral problems, and emotional reactions. Each item is scored on a 7-point scale (ranging from 1 to 7), with higher total scores indicating poorer quality of life [5].

Pediatric Sleep Questionnaire (PSQ): This questionnaire is commonly used worldwide as a screening tool for pediatric OSA and can effectively identify children at high risk of OSA [8,9,10,11,12]. A total PSQ score >7 was used as the threshold for identifying children at high risk of OSA, based on established criteria [11,12,13,14]. It is worth noting that all analyses and conclusions related to OSA in this study refer specifically to the risk status determined by the PSQ, rather than a formal diagnosis confirmed by PSG.

**Figure 1:**
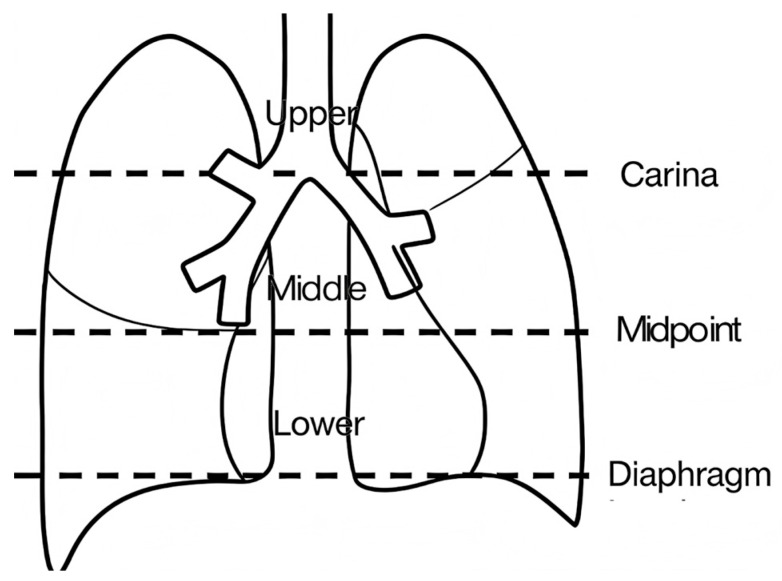
Pulmonary imaging segmentation.

Pulmonary Imaging Score: Visual semi-quantitative scoring was used in this study to evaluate the pulmonary images of the children. The lung field segmentation was performed according to the method of Saraya et al. [15]. Each hemithorax was divided longitudinally into three parts based on the carina, the level of the diaphragm, and the midpoint between them, resulting in a total of 6 zones (Figure 1). The severity of lesions was assessed using the dual-dimension scoring principle of “region and severity”, which is widely used for evaluating pneumonia severity [16,17,18]. Each zone was scored 0–3 according to the extent of involvement and 0–2 according to the severity of lesions, with a total score of 30 (Table 1).

### 2.3. Data Collection

The following data were collected from all participants: (1) General information: sex, age, height, weight, body mass index (BMI), gestational age at birth, mode of delivery, birth weight, and history of corticosteroid use prior to blood collection. (2) Clinical data: history of allergic diseases, history of wheezing, fever duration, peak fever temperature, cough duration, pulmonary imaging findings (all patients underwent chest radiography or CT examination), macrolide antibiotic use and second-line agent selection, corticosteroid therapy, oxygen therapy, and bronchoscopic bronchoalveolar lavage (BAL) treatment. (3) Laboratory parameters: All laboratory indicators were measured within 24 h of admission, including white blood cell count (WBC), eosinophil count (EOS) and percentage (EOS%), procalcitonin (PCT), C-reactive protein (CRP), MP-IgM, MP-DNA or MP-RNA, lactate dehydrogenase (LDH), D-dimer (D-D), ferritin, alanine aminotransferase (ALT), aspartate aminotransferase (AST), heparin-binding protein (HBP), lymphocyte subsets (CD4^+^, CD8^+^, CD4^+^/CD8^+^ ratio), interferon-α (IFN-α), tumor necrosis factor-α (TNF-α), and interleukins (IL-2, IL-4, IL-5, IL-6, IL-10, IL-17). (4) Questionnaire assessments: Both questionnaires were administered by trained, designated personnel. PSQ was completed for all participants, and RQLQ was completed for children aged 6–12 years complicated with AR.

### 2.4. Statistical Analysis

Statistical analysis was performed using SPSS version 27.0. Normally distributed continuous variables were expressed as mean ± standard deviation and compared between groups using the independent samples *t*-test. Non-normally distributed continuous variables were expressed as median (Q1, Q3) and compared using the Mann–Whitney U test. Categorical variables were presented as rates (%) and compared using the chi-square (*χ*^2^) test or corrected *χ*^2^ test. Spearman correlation analysis was used to assess correlations between variables, with a correlation coefficient of *r* < 0.5 indicating no significant collinearity. Univariate logistic regression analysis was performed to screen for potential risk factors (inclusion criterion: *p* < 0.1). Backward elimination was then applied to sequentially remove non-significant variables (exclusion criterion: *p* > 0.1). Variables retained from this process were entered into multivariate logistic regression analysis to identify independent risk factors for SMPP. A nomogram prediction model was subsequently constructed using R software (version 4.5.2). The predictive value of individual risk factors and the nomogram for SMPP was evaluated using receiver operating characteristic (ROC) curve analysis. The optimal cutoff values for LDH and ferritin were determined using the Youden index based on the receiver operating characteristic (ROC) curve. Internal validation of the nomogram was performed using the Bootstrap resampling. Calibration was assessed using calibration curves and the Hosmer–Lemeshow goodness-of-fit test. Clinical utility was evaluated using decision curve analysis (DCA). A two-tailed *p* < 0.05 was considered statistically significant.

## 3. Results

### 3.1. Comparison of General Information Between Groups

Among the 150 enrolled children with MPP, 35 were classified into the SMPP group and 115 into the MMPP group. In the SMPP group, the mean age was 8.76 ± 2.60 years, with 17 males and 18 females. In the MMPP group, the mean age was 7.91 ± 2.90 years, with 56 males and 59 females. There were no significant differences between the two groups in gender, age, gestational age at birth, mode of delivery, birth weight, BMI, or history of corticosteroid use prior to blood collection (all *p* > 0.05, Table 2).

### 3.2. Comparison of Clinical Data Between Groups

In the SMPP group, 22 children (62.9%) had comorbid AD, significantly higher than 49 cases (42.6%) in the MMPP group (*χ*^2^ = 4.41, *p* = 0.036). There were no significant differences between the two groups in the proportion of children with a history of AR or history of wheezing (*p* > 0.05). The SMPP group exhibited significantly higher peak fever temperature (*t* = 2.25, *p* = 0.026), pulmonary imaging scores (*t* = 10.69, *p* < 0.001), and duration of systemic corticosteroid use (*Z* = 2.84, *p* = 0.005) compared to the MMPP group. No significant differences were found in fever duration or cough duration between groups (*p* > 0.05). (Table 3)

### 3.3. Comparison of Laboratory Parameters Between Groups

In the SMPP group, levels of LDH, D-dimer, ferritin, and ALT were significantly higher than those in the MMPP group (*Z* = 2.70, *p* = 0.007; *Z* = 2.87, *p* = 0.004; *Z* = 2.70, *p* = 0.007; *Z* = 2.13, *p* = 0.034). CRP also showed a borderline elevation in the SMPP group (*Z* = 1.96, *p* = 0.050). No significant differences were observed in the other parameters (*p* > 0.05). (Table 4).

### 3.4. RQLQ Scores in MPP Children Aged 6–12 Years Complicated with AR

Children who developed SMPP had significantly higher RQLQ scores compared to those with MMPP, indicating poorer AR-related quality of life (*Z* = 2.03, *p* = 0.042). There were no significant differences in the remaining domain scores between the two groups (all *p* > 0.05). (Table 5)

### 3.5. PSQ Scores in Children with MPP

The SMPP group exhibited significantly higher total PSQ scores, as well as higher scores in the respiratory, sleep, and behavioral domains compared to the MMPP group (*Z* = 4.00, *p* < 0.001; *Z* = 2.65, *p* = 0.008; *Z* = 1.98, *p* = 0.048; *Z* = 2.64, *p* = 0.008, Table 6). The proportion of children with PSQ score >7 was significantly higher in the SMPP group than in the MMPP group (17.1% vs. 4.3%, *χ*^2^ = 5.45, *p* = 0.020, Table 6).

### 3.6. Spearman Correlation Analysis

**Figure 2:**
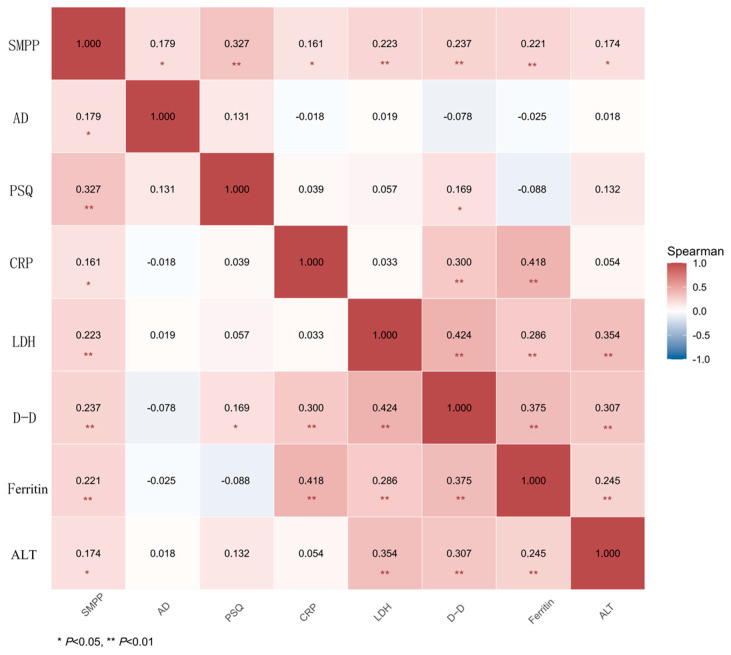
Spearman correlation analysis of clinical information and SMPP.

Spearman correlation analysis revealed that MPP severity was positively correlated with AD (*r* = 0.179, *p* = 0.028), PSQ score (*r* = 0.327, *p* < 0.001), CRP (*r* = 0.161, *p* = 0.049), LDH (*r* = 0.223, *p* = 0.006), D-dimer (*r* = 0.237, *p* = 0.004), ferritin (*r* = 0.221, *p* = 0.007), and ALT (*r* = 0.174, *p* = 0.036). All correlation coefficients among continuous variables were less than 0.5, indicating no significant collinearity (Figure 2). Among children aged 6–12 years with comorbid AR, MPP severity was also positively correlated with RQLQ score (*r* = 0.299, *p* = 0.041).

### 3.7. Univariate Logistic Regression Analysis

Univariate logistic regression analysis showed that AD, PSQ score, CRP, LDH, D-dimer, ferritin, and ALT were significantly associated with SMPP (all *p* < 0.05). The specific odds ratios (*OR*) and 95% confidence intervals (*CI*) are presented in Table 7.

### 3.8. Multivariate Logistic Regression Analysis and Predictive Nomogram

**Figure 3:**
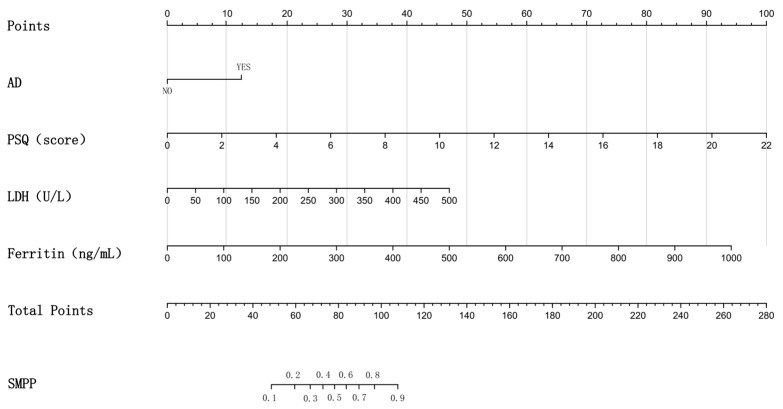
Nomogram for risk prediction of SMPP.

Univariate logistic regression identified seven variables associated with SMPP at *p* < 0.1: AD, PSQ score, CRP, LDH, D-dimer, ferritin, and ALT. Using backward stepwise regression, the multivariate logistic regression model ultimately identified AD, PSQ score, LDH, and ferritin as independent risk factors for SMPP (Table 8): AD (*OR* = 2.507, 95% CI: 1.008–6.234, *p* = 0.048), PSQ score (*OR* = 1.403, 95% CI: 1.177–1.672, *p* < 0.001), LDH (*OR* = 1.007, 95% CI: 1.001–1.013, *p* = 0.029), and ferritin (*OR* = 1.007, 95% CI: 1.001–1.014, *p* = 0.018). Based on these results, a nomogram prediction model was constructed by combining AD, PSQ score, LDH, and ferritin (Figure 3).

### 3.9. ROC Curve Analysis for Predicting SMPP

**Figure 4:**
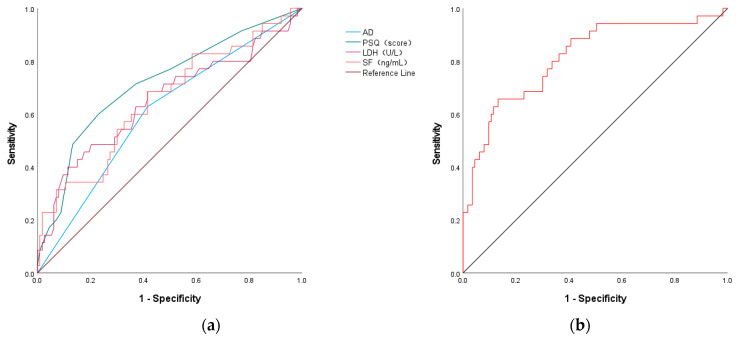
(**a**) ROC curve analysis of independent risk factors in predicting SMPP; (**b**) ROC curve of the nomogram for SMPP risk prediction.

AD, PSQ, LDH, and ferritin showed certain predictive value for the occurrence of SMPP. The area under the curve (AUC) was 0.606 (95% CI: 0.500–0.713, *p* = 0.058), 0.720 (95% CI: 0.617–0.820, *p* < 0.001), 0.652 (95% CI: 0.538–0.766, *p* = 0.007), and 0.653 (95% CI: 0.545–0.760, *p* = 0.006), respectively. The optimal cut-off value for PSQ score was 3.5, with a sensitivity of 60.0% and specificity of 77.4%. The optimal cut-off value for LDH was 327.50 U/L, with a sensitivity of 40.0% and specificity of 88.7%. The optimal cut-off value for ferritin was 120.05 ng/mL, with a sensitivity of 68.6% and specificity of 57.4% (Table 9 and Figure 4a). The nomogram constructed by combining the above independent risk factors demonstrated an area under the curve (AUC) of 0.814 (95% CI: 0.726–0.901, *p* < 0.001) for predicting SMPP occurrence (Figure 4b), with a sensitivity of 65.7% and specificity of 86.7%.

### 3.10. Validation of the Nomogram

**Figure 5:**
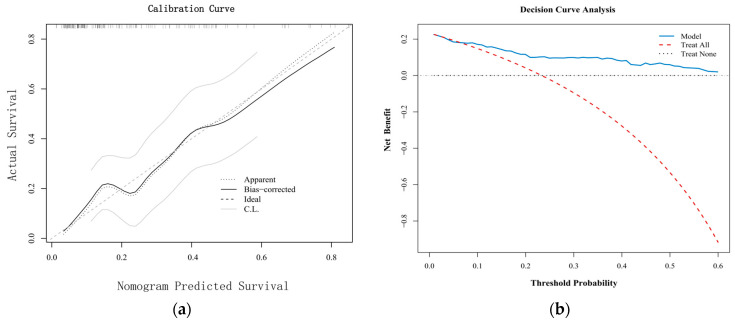
(**a**) Calibration Curve. (**b**) Decision Curve Analysis.

Internal validation of the model was performed using the Bootstrap resampling method (1000 resamples). The original C-index of the nomogram was 0.815, and the Bootstrap-corrected C-index was 0.827, with an optimism of −0.012, indicating no significant overfitting. The Hosmer–Lemeshow test (*χ*^2^ = 12.38, *p* = 0.116) and the calibration curve (Figure 5a) collectively confirmed good calibration of the nomogram.

Decision curve analysis demonstrated that within a threshold probability range of 0–25%, the decision strategy based on this nomogram provided a superior net benefit compared to the extreme strategies of “intervene for all” or “intervene for none”. The maximum net benefit was achieved at a threshold probability of 10% (net benefit = 0.09), corresponding to avoiding 90 unnecessary interventions per 1000 patients (Figure 5b).

## 4. Discussion

*Mycoplasma pneumoniae* (MP) is a common and special pathogen causing respiratory tract infections in children in China. *Mycoplasma pneumoniae* pneumonia (MPP) accounts for 10% to 40% of community-acquired pneumonia (CAP) cases among children aged 5 years and older in China, and school-age children aged 7 to 12 years are at high risk of MP infection [1,19]. A study from the United States similarly reported that children aged 6–12 years had the highest hospitalization rate for MPP (42.6%) [20]. In some cases, the infection can progress to severe *Mycoplasma pneumoniae* pneumonia (SMPP) and may even lead to serious conditions such as necrotizing pneumonia, bronchiolitis obliterans, and thrombosis. These conditions can cause severe extrapulmonary complications and result in a major medical burden [21]. In 2022, the proportion of children with MPP progressing to SMPP in Hangzhou, China, reached as high as 38.3% [19].

The pathogenesis of MPP remains incompletely understood. Most current studies suggest that the pathogenesis involves direct damage caused by MP and abnormal immune responses of the host. The severity of MPP depends on the interaction between the host and MP [22,23,24,25]. Increasing attention has been directed toward the impact of host factors on the severity of MP infection. Studies have shown that allergic diseases (AD) may exacerbate the condition of children with MPP and increase the risk of extrapulmonary complications [26]. A study investigating risk factors for recurrent respiratory tract infections in children found that children with a history of allergic rhinitis (AR) were more likely to experience recurrent respiratory tract infections compared to healthy controls [27]. Obstructive sleep apnea (OSA) is characterized by sleep fragmentation and chronic intermittent hypoxia, leading to airway hyperresponsiveness and increased levels of inflammatory cytokines, which adversely affect immune function. In severe cases, OSA may result in hypercapnia, impair pulmonary neutrophil function, and predispose patients to lower respiratory tract infections [28,29,30,31,32]. Another study has shown that OSA is closely related to the severity of CAP [7]. Therefore, the presence of AD or OSA may be important host background that promotes the progression to SMPP in children after MP infection.

This study suggested that the prevalence of AD was significantly higher in the SMPP group compared to the MMPP group (*p* < 0.05). Multivariate logistic regression analysis identified AD as an independent risk factor for SMPP, consistent with findings reported by Qin et al. [33,34,35]. As an intracellular pathogen capable of long-term survival under certain conditions [36], MP is eliminated mainly through the activation of macrophages, natural killer (NK) cells, and CD8+ T cells, which is mediated by Th1 immune responses [37]. Yao et al. [38] reported that children with MPP and a background of AD exhibit a significant Th2 immune deviation, characterized by more active Th2-type inflammation and relative suppression of Th1-type responses. Consequently, children with MPP and an AD background were more likely to develop severe disease.

In our study, levels of CRP, LDH, ferritin, D-dimer, and ALT were significantly higher in the SMPP group compared to the MMPP group, consistent with previous studies [39,40,41,42]. Spearman correlation analysis revealed that the degree of elevation in these indicators was positively correlated with the severity of MPP. Multivariate logistic regression analysis identified LDH and ferritin as independent risk factors for SMPP. Our results showed that LDH > 327.5 U/L predicted SMPP with a sensitivity of 40.0% and specificity of 88.7%, while ferritin > 120.05 ng/mL predicted SMPP with a sensitivity of 68.6% and specificity of 57.4%, suggesting that elevated LDH or ferritin beyond these thresholds may warrant clinical vigilance for SMPP. As a key enzyme in the glycolytic pathway, LDH is widely present in the cytoplasm of various tissue cells in the human body. It participates in cellular energy metabolism by catalyzing the conversion between lactate and pyruvate. Among its isoenzymes, LDH4 and LDH5 are primarily derived from lung tissue. When organs undergo inflammatory damage, particularly when lung tissue is compromised due to hypoxia, LDH is released into the bloodstream and extracellular space as a result of cell division or cell membrane damage, leading to increased LDH levels in peripheral blood [43]. Therefore, LDH is commonly used as a nonspecific marker of tissue injury and cell death to assess disease severity and prognosis [44]. Ferritin serves as a key intracellular iron storage protein and is also one of the acute-phase reactants. It is released by hepatocytes and macrophages in response to proinflammatory cytokine stimulation. Elevated ferritin levels reflect macrophage activation and an excessive inflammatory response in the body [43]. In our study, both LDH and ferritin were elevated in children with SMPP and were identified as independent risk factors for SMPP, suggesting that lung tissue damage and excessive immune-inflammatory responses may jointly contribute to the pathogenesis of SMPP.

Cellular immunity mediated by Th1 cells helps clear intracellular pathogens. However, an excessively activated Th1 response can aggravate inflammation by releasing large amounts of proinflammatory cytokines, which eventually leads to immune damage in the body [37]. Studies have shown that the cytotoxic molecule CARDS TX secreted by MP can promote the differentiation of Th1 cells by stimulating dendritic cells (DCs) to produce IFN-γ. IFN-γ further induces M1 macrophages to secrete chemokine ligand 9 (CXCL9) through the STAT1 signaling pathway, which promotes the migration of Th1 cells. This forms a positive feedback loop of the Th1 immune response and amplifies inflammatory injury in the lungs [45,46]. A study by Jia et al. [47] also confirmed that Th1 cells are overactivated after MP infection, and the intensity of the Th1 response is positively correlated with the severity of MPP. IL-2 is mainly secreted by Th1 cells. Bao et al. [48] found that the peripheral blood IL-2 level was increased in the SMPP group, which indirectly supports the overactivation of Th1 cells. However, Esposito et al. [49] found no significant difference in serum IL-2 levels between children with MP infection and healthy controls, which is consistent with the results of this study. Bao et al. [48] suggested that such differences may be related to factors including age, infection severity, and baseline immune status of the children. Therefore, it is speculated that children with SMPP and a background of AD may have a complex immune status characterized by the coexistence of allergy-related Th2 dominance and infection-related Th1 activation. Thus, the level of IL-2 may be affected by multiple factors such as sampling time, disease stage, and immune status. Further large-scale and homogeneous clinical studies are needed to clarify the clinical significance of IL-2 measurement.

In this study, there was no significant difference in the prevalence of AR between the two groups (*p* > 0.05). To further explore the relationship between AR-related quality of life and the development of SMPP, the RQLQ scale was used to evaluate children aged 6–12 years with MPP and AR. The results showed that the RQLQ score was significantly higher in the SMPP group than in the MMPP group (*p* < 0.05), indicating worse AR-related quality of life. Spearman correlation analysis demonstrated that the severity of MPP was positively correlated with the RQLQ score. These findings suggest that children with poorer AR-related quality of life are more likely to progress to SMPP. However, this analysis was based on a relatively small subgroup (*n* = 47), and these findings should be interpreted with caution.

Polysomnography (PSG) is the gold standard for the diagnosis of OSA [6]. However, PSG requires professional sleep laboratories, equipment, and trained personnel for result interpretation. It is limited in population screening due to high cost, complex operation, poor cooperation in children, and low convenience [50]. In addition, the obstructive apnea/hypopnea index (OAHI) has a weak correlation with clinical manifestations and cannot evaluate daytime symptoms and quality of life in children [51]. Therefore, this study used the PSQ to screen children at high risk of OSA. This questionnaire is commonly used worldwide as a screening tool for pediatric OSA and can effectively identify children at high risk of OSA [8,9,10,11,12]. Based on this, all analyses and conclusions related to OSA in this study refer specifically to the risk status determined by the PSQ, rather than a formal diagnosis confirmed by PSG. In this study, the total PSQ score and scores of domains including respiration, sleep, and behavior were higher in the SMPP group than in the MMPP group, and the proportion of children at high risk of OSA was also significantly higher (*p* < 0.05). Spearman correlation analysis showed that the severity of MPP was positively correlated with the PSQ score. Multivariate Logistic regression analysis indicated that the PSQ score was an independent risk factor for SMPP. Studies by Atceken [52] and Wang et al. [53] both suggested that OSA may aggravate the severity of respiratory infections through common mechanisms. Although the exact mechanisms remain to be fully elucidated, several potential pathways have been proposed. The core pathophysiology of OSA is repeated upper airway collapse during sleep, leading to chronic intermittent hypoxia (CIH), hypercapnia, and sleep fragmentation [54]. When MP infects children with underlying CIH, the oxidative stress induced by CIH and the direct airway epithelial injury caused by MP are superimposed, which further aggravates pulmonary inflammation. Meanwhile, hypercapnia caused by CIH can reduce the levels of TNF-α and IL-8 and affect the recruitment and activation of neutrophils [55,56,57], thus weakening the body’s ability to clear MP. In children with OSA, adenoid and tonsillar hypertrophy are the main anatomical factors. A study by Li et al. [58] found that persistent tonsillar hypertrophy is often accompanied by cell senescence and chronic inflammatory cell infiltration. The mechanism is related to a cycle of persistent infection, tissue injury, and pathological repair caused by impaired pathogen clearance [59]. In addition, bony abnormalities such as micrognathia and narrow pharyngeal cavity can reduce the clearance of secretions and promote MP colonization. Increased intrathoracic pressure after apnea in patients with OSA may cause aspiration of pathogen-laden pharyngeal secretions into the lower respiratory tract [60,61]. Meanwhile, OSA is often accompanied by gastroesophageal reflux, and the reflux of gastric contents can further irritate and damage the lower respiratory tract, increasing the risk and severity of infection [62]. In conclusion, OSA may aggravate the severity of respiratory infections through the above mechanisms, and children with MPP who are at high risk of OSA are more likely to develop SMPP.

The nomogram model established in our study integrated four indicators: history of allergic diseases, PSQ score, LDH, and ferritin. The predictive performance of the model was verified through multi-dimensional validation. The AUC for predicting the occurrence of SMPP was 0.814, indicating good discrimination. The calibration curve showed that the predicted probability of the model was highly consistent with the actual risk. Decision curve analysis confirmed that the model had favorable clinical net benefit. All indicators in the model can be obtained through medical history collection, questionnaire evaluation, and routine biochemical tests, allowing early risk assessment during the course of the disease. Therefore, this model has both stable predictive performance and high clinical practicability, making it an optimal tool for the early identification and risk stratification of SMPP. In clinical practice, when a child presents with M. pneumoniae infection, the clinician can easily obtain the four predictors (history of allergic diseases, PSQ score, LDH, and ferritin) as part of routine evaluation. The nomogram then provides an estimated probability of progressing to severe disease. This tool would be most beneficial for children at the early stage of illness, where risk stratification is challenging but clinically important. For those identified as high risk, we suggest closer monitoring. This may include daily follow-up, early hospitalization, or more frequent vital sign checks. A lower threshold for initiating supportive therapies should also be considered. For low-risk children, the nomogram may reassure clinicians and families, helping to avoid unnecessary hospital admissions or overtreatment.

However, this study was retrospective and single-center in design, with a moderate sample size that included only 35 SMPP cases among 150 patients. Although our analysis yielded promising results, these factors may introduce selection bias and limit the generalizability of the findings. Therefore, our proposed nomogram requires prospective, multicenter external validation before its broad applicability can be assumed. Readers are advised to interpret the model’s performance with caution.

## 5. Conclusions

In this retrospective and single-center study, children with MPP who have AD or OSA risk appeared to be more susceptible to developing SMPP. Among children aged 6–12 years, RQLQ score is positively correlated with MPP severity. AD, PSQ score, LDH, and ferritin were identified as independent risk factors for SMPP. For children with MPP, the development of SMPP should be closely monitored if they have a history of AD, PSQ score > 3.5, LDH > 327.50 U/L, or ferritin > 120.05 ng/mL. The nomogram visualization model constructed by combining these risk factors showed promising predictive performance for SMPP, with high predictive efficacy and accuracy, suggesting potential clinical utility for individualized risk assessment and early intervention. This study has several limitations. First, the single-center retrospective design with a limited sample size may introduce selection bias. Therefore, given the moderate sample size and the lack of external validation, these findings are preliminary. Second, although no statistically significant difference was observed between groups regarding outpatient corticosteroid use prior to admission, this factor may still have potential effects on the immune status of the children. Specifically, corticosteroids could suppress systemic inflammatory markers such as LDH and ferritin, or alter clinical presentations such as fever and respiratory symptoms at admission, thereby potentially influencing disease severity classification and model performance. Third, only internal validation was performed without external validation. Therefore, the conclusions of this study need to be further verified through prospective, multicenter studies with larger sample sizes before the nomogram can be recommended for routine clinical use. Fourth, OSA risk was assessed using the PSQ, a screening tool rather than a diagnostic standard for OSA. Our findings therefore refer to PSQ-defined risk status rather than PSG-confirmed diagnosis.

## Figures and Tables

**Table 1:** Pulmonary imaging scoring criteria.

Scoring Criteria	Pulmonary Imaging Manifestations
Extent of involvement	0 point: Normal
	1 point: <25%
	2 points: 25–50%
	3 points: >50%
Severity of lesion	0 point: Normal
	1 point: Ground-glass opacities, hazy increased density with visible bronchial and vascular markings
	2 points: Consolidation, homogeneous opacification with obliteration of pulmonary vessels and airway walls, air bronchograms may be visible

**Table 2:** Comparison of general information between SMPP group and MMPP group.

General Information	SMPP (*n* = 35)	MMPP (*n* = 115)	*t*/*χ*^2^/*Z* Value	*p* Value
Gender			0.00	0.990
Male, *n* (%)	17 (48.6)	56 (48.7)		
Female, *n* (%)	18 (51.4)	59 (51.3)		
Age (year)	8.76 ± 2.60	7.91 ± 2.90	1.56	0.121
Gestational age at birth			1.25	0.573
Term, *n* (%)	35 (100.0)	111 (96.5)		
Preterm, *n* (%)	0 (0.0)	4 (3.5)		
Mode of delivery			0.89	0.347
Vaginal, *n* (%)	16 (45.7)	63 (54.8)		
Cesarean, *n* (%)	19 (54.3)	52 (45.2)		
Birth weight (kg)	3.24 ± 0.44	3.30 ± 0.49	−0.66	0.513
BMI (kg/m^2^)	16.17 ± 2.84	16.34 ± 2.87	−0.32	0.752
Corticosteroid use prior to blood collection			1.61	0.205
Yes, *n* (%)	16 (45.7)	39 (33.9)		
No, *n* (%)	19 (54.3)	76 (66.1)		
Duration of corticosteroid use prior to blood collection (days)	0.00 (0.00, 1.00)	0.00 (0.00, 1.00)	−0.82	0.411

**Table 3:** Comparison of clinical data between SMPP group and MMPP group.

Clinical Data	SMPP (*n* = 35)	MMPP (*n* = 115)	*χ*^2^/*t*/*Z* Value	*p* Value
AD			4.41	0.036
Yes, *n* (%)	22 (62.9)	49 (42.6)		
No, *n* (%)	13 (37.1)	66 (57.4)		
AR			0.09	0.763
Yes, *n* (%)	15 (42.9)	46 (40.0)		
No, *n* (%)	20 (57.1)	69 (60.0)		
History of wheezing			0.06	0.802
Yes, *n* (%)	8 (22.9)	24 (20.9)		
No, *n* (%)	27 (77.1)	91 (79.1)		
Fever duration (days)	5.77 ± 2.79	5.24 ± 2.83	0.98	0.332
Peak fever temperature (°C)	39.53 ± 0.59	39.25 ± 0.64	2.25	0.026
Cough duration (days)	11.60 ± 3.96	11.97 ± 4.93	−0.46	0.647
Pulmonary imaging scores (points)	9.46 ± 2.417	4.87 ± 2.162	10.69	<0.001
Corticosteroid use			3.33	0.068
Yes, *n* (%)	28 (80.0)	73 (63.5)		
No, *n* (%)	7 (20.0)	42 (36.5)		
Total duration of corticosteroid use (d)	9.00 (4.00, 12.00)	4.00 (0.00, 9.00)	2.84	0.005

**Table 4:** Comparison of laboratory parameters between SMPP group and MMPP group.

Laboratory Parameters	SMPP (*n* = 35)	MMPP (*n* = 115)	*Z* Value	*p* Value
WBC (×10^9^/L)	7.01 (4.86, 8.61)	7.16 (5.50, 9.37)	−0.56	0.574
EOS (%)	0.50 (0.20, 1.00)	0.90 (0.20, 2.50)	−1.31	0.190
EOS (×10^9^/L)	0.04 (0.02, 0.16)	0.07 (0.02, 0.18)	−1.03	0.306
CRP (mg/L)	20.10 (7.10, 30.90)	9.80 (5.80, 22.70)	1.96	0.050
SAA (mg/L)	163.12 (56.60, 338.18)	84.90 (23.20, 211.10)	1.65	0.100
PCT (ng/mL)	0.20 (0.15, 0.35)	0.19 (0.13, 0.29)	1.30	0.195
LDH (U/L)	297.00 (251.00, 364.00)	263.00 (230.00, 299.00)	2.70	0.007
D-D (mg/L)	0.23 (0.14, 0.34)	0.15 (0.10, 0.22)	2.87	0.004
Ferritin (ng/mL)	140.4 (105.20, 199.60)	110.2 (86.20, 151.00)	2.70	0.007
ALT (U/L)	14.00 (12.00, 25.00)	12.00 (11.00, 16.00)	2.13	0.034
AST (U/L)	27.00 (23.00, 34.00)	25.00 (21.00, 31.00)	1.69	0.091
HBP (ng/mL)	51.75 (28.25, 87.00)	57.73 (30.58–98.13)	−0.55	0.583
CD4^+^ (cells/mm^3^)	402.00 (220.38, 614.63)	506.82 (358.31, 772.39)	−1.93	0.054
CD8^+^ (cells/mm^3^)	320.00 (198.45, 405.12)	360.95 (255.00, 567.10)	−1.75	0.080
CD4^+^/CD8^+^	1.34 (1.00, 1.90)	1.33 (1.10, 1.85)	−0.44	0.663
INF-γ (pg/mL)	8.70 (6.80, 17.70)	8.30 (6.10, 11.90)	1.12	0.261
TNF-α(pg/mL)	3.40 (2.80, 4.50)	3.50 (2.90, 4.20)	−0.30	0.763
IL-2 (pg/mL)	3.80 (3.40, 4.20)	3.80 (3.40, 4.30)	−0.16	0.874
IL-4 (pg/mL)	4.20 (3.90, 5.20)	4.20 (3.60, 5.60)	0.17	0.864
IL-5 (pg/mL)	4.80 (3.00, 8.30)	4.00 (2.90, 6.20)	0.88	0.377
IL-6 (pg/mL)	28.40 (11.80, 104.20)	20.10 (8.59, 59.30)	0.99	0.322
IL-10 (pg/mL)	7.20 (2.80, 9.00)	7.10 (4.79, 8.60)	−0.09	0.926
IL-17 (pg/mL)	3.00 (1.77, 6.70)	3.40 (2.01, 6.50)	−0.62	0.539

**Table 5:** Comparison of RQLQ between groups *.

RQLQ Scores	SMPP (*n* = 13)	MMPP (*n* = 34)	*Z* Value	*p* Value
Total (score)	45.00 (35.50, 50.00)	36.00 (33.00, 45.25)	2.03	0.042
Nose symptoms (score)	12.00 (8.00, 12.50)	10.00 (7.75, 12.00)	0.96	0.336
Eye symptoms (score)	7.00 (4.50, 8.50)	5.00 (4.00 6.25)	1.79	0.073
Other symptoms (score)	13.00 (9.50, 17.00)	10.00 (9.00, 12.25)	1.70	0.089
Behavioral problems (score)	10.00 (7.50, 13.00)	8.50 (6.00, 10.00)	1.77	0.077
Emotional reactions (score)	3.00 (2.00, 5.50)	2.50 (2.00, 4.00)	0.79	0.430

* This table only includes children aged 6–12 years with comorbid AR. This analysis was performed on a relatively small subgroup of patients (*n* = 47), which limits the generalizability of the conclusions. Future studies with larger sample sizes are needed to validate these findings.

**Table 6:** Comparison of PSQ between groups.

PSQ	SMPP (*n* = 35)	MMPP (*n* = 115)	*Z/χ*^2^ Value	*p* Value
Total (score)	4.00 (2.00, 5.00)	1.00 (1.00, 3.00)	4.00	<0.001
Respiratory (score)	1.00 (0.00, 2.00)	0.00 (0.00, 1.00)	2.65	0.008
Sleep (score)	0.00 (0.00, 0.00)	0.00 (0.00, 0.00)	1.98	0.048
Behavioral (score)	1.00 (0.00, 4.00)	0.00 (0.00, 2.00)	2.64	0.008
Other (score)	1.00 (0.00, 1.00)	0.00 (0.00, 1.00)	1.94	0.053
Score > 7			5.45	0.020
Yes, *n* (%)	6 (17.1)	5 (4.3)		
No, *n* (%)	29 (82.3)	110 (95.7)		

**Table 7:** Results of univariate logistic regression analysis.

Variables	*B*	SE	Wald *χ*^2^	*OR*	95% CI	*p* Value
AD	0.860	0.398	4.672	2.362	1.083–5.150	0.031
Total PSQ (score)	0.316	0.081	15.351	1.371	1.171–1.606	<0.001
CRP (mg/L)	0.016	0.008	4.006	1.017	1.000–1.033	0.045
LDH (U/L)	0.008	0.003	8.776	1.008	1.003–1.014	0.003
D-D (mg/L)	2.151	1.078	3.980	8.594	1.039–71.104	0.046
Ferritin (ng/mL)	0.008	0.003	7.989	1.008	1.002–1.014	0.005
ALT (U/L)	0.057	0.024	5.895	1.059	1.011–1.109	0.015

**Table 8:** Results of multivariate logistic regression analysis.

Variables	*B*	SE	Wald *χ*^2^	*OR*	95% CI	*p* Value
AD	0.919	0.465	3.908	2.507	1.008–6.234	0.048
Total PSQ (score)	0.338	0.090	14.243	1.403	1.177–1.672	<0.001
LDH (U/L)	0.007	0.003	4.767	1.007	1.001–1.013	0.029
Ferritin (ng/mL)	0.007	0.003	5.553	1.007	1.001–1.014	0.018

**Table 9:** The predictive efficacy of each independent risk factor in predicting SMPP.

Independent Risk Factors	AUC	95% CI	*p* Value	Optimal Cut-Off	Sensitivity	Specificity
AD	0.606	0.500–0.713	0.058	0.5	62.9%	58.3%
Total PSQ (score)	0.720	0.617–0.820	<0.001	3.5	60.0%	77.4%
LDH (U/L)	0.652	0.538–0.766	0.007	327.50	40.0%	88.7%
Ferritin (ng/mL)	0.653	0.545–0.760	0.006	120.05	68.6%	57.4%

## Data Availability

The data presented in this study are available upon request from the corresponding author due to ethical restrictions.

## Abbreviations

The following abbreviations are used in this manuscript:

AD	Allergic diseases
ALT	Alanine aminotransferase
AR	Allergic rhinitis
AST	Aspartate aminotransferase
AUC	Area under the curve
BMI	Body Mass Index
CAP	Community-Acquired Pneumonia
CD4^+^	CD4-positive T cell
CD8^+^	CD8-positive T cell
CI	Confidence Interval
CIH	Chronic Intermittent Hypoxia
CRP	C-reactive protein
CT	Computed Tomography
DCA	Decision Curve Analysis
D-D	D-dimer
EOS	Eosinophil
MMPP	Mild *Mycoplasma pneumoniae* Pneumonia
HBP	Heparin-binding Protein
IL	Interleukin
IFN	Interferon
LDH	Lactate dehydrogenase
MP	*Mycoplasma pneumoniae*
MPP	*Mycoplasma pneumoniae* Pneumonia
OAHI	Obstructive Apnea/Hypopnea Index
OR	Odds ratio
OSA	Obstructive Sleep Apnea
PCT	Procalcitonin
PRQLQ	Pediatric Rhinoconjunctivitis Quality of Life Questionnaire
PSG	Polysomnography
PSQ	Pediatric Sleep Questionnaire
ROC	Receiver Operating Characteristic curve
SMPP	Severe *Mycoplasma pneumoniae* Pneumonia
Th1	Helper T lymphocytes 1
Th2	Helper T lymphocytes 2
TNF	Tumor Necrosis Factor
WBC	White blood cell count

## References

[1] Sun Y., Li P., Jin R., Liang Y., Yuan J., Lu Z., Liang J., Zhang Y., Ren H., Zhang Y. (2025). Characterizing the epidemiology of *Mycoplasma pneumoniae* infections in China in 2022–2024: A nationwide cross-sectional study of over 1.6 million cases. Emerg. Microbes Infect..

[2] Yan C., Xue G.-H., Zhao H.-Q., Feng Y.-L., Cui J.-H., Yuan J. (2024). Current status of *Mycoplasma pneumoniae* infection in China. World J. Pediatr..

[3] Paquette M., Magyar M., Renaud C. (2024). *Mycoplasma* *pneumoniae*. Can. Med. Assoc. J..

[4] National Health Commission, National Administration of Traditional Chinese Medicine (2025). Guidelines for the diagnosis and treatment of *Mycoplasma pneumoniae* pneumonia in children (2025 edition). Chin. J. Evid. Based Pediatr..

[5] (2022). Subspecialty Group of Rhinology; Editorial Board of Chinese Journal of Otorhinolaryngology Head and Neck Surgery; Subspecialty Groups of Rhinology and Pediatrics; Society of Otorhinolaryngology Head and Neck Surgery, Chinese Medical Association. Guideline for diagnosis and treatment of pediatric allergic rhinitis (2022, revision). Chin. J. Otorhinolaryngol. Head Neck Surg..

[6] Working Group of Chinese Guideline for the Diagnosis and Treatment of Childhood OSA, Subspecialty Group of Pediatrics, Society of Otorhinolaryngology Head and Neck Surgery, Chinese Medical Association, Subspecialty Group of Respiratory Diseases, Society of Pediatrics, Chinese Medical Association, Society of Pediatric Surgery, Chinese Medical Association, Editorial Board of Chinese Journal of Otorhinolaryngology Head and Neck Surgery (2020). Chinese guideline for the diagnosis and treatment of childhood obstructive sleep apnea (2020). Chin. J. Otorhinolaryngol. Head Neck Surg..

[7] Chiner E., Llombart M., Valls J., Pastor E., Sancho-Chust J.N., Andreu A.L., Sánchez-de-la-Torre M., Barbé F. (2016). Association between Obstructive Sleep Apnea and Community-Acquired Pneumonia. PLoS ONE.

[8] Spruyt K., Gozal D. (2011). Pediatric sleep questionnaires as diagnostic or epidemiological tools: A review of currently available instruments. Sleep Med. Rev..

[9] Masoud A.I., Adavadkar P.A., Park C., Gowharji L.F., Alwadei A.H., Carley D.W. (2022). Comparing two pediatric sleep questionnaires: The Pediatric Sleep Questionnaire (PSQ) and a set of 6 hierarchically arranged questions (6Q). Cranio.

[10] Bseikri M., Zhang J., Kirley J., Lee C., Castillo A., Feliciano E.M.C. (2023). Predicting obstructive sleep apnea severity in children referred for polysomnography: Use of the Pediatric Sleep Questionnaire and Subscales. Sleep Breath..

[11] Wang Y.Y., Meng L.P., Ji H., Liu M., Hong Q. (2023). Application of OSA-18 and PSQ in preschool children with OSA. Chin. Arch. Otolaryngol. Head Neck Surg..

[12] Tai J., Xu Z.F., Li X.D., Du J.N., Wang G.X., Ma J., Hu P.J., Yan X.Y., Zhang J., Zhang Y.M. (2018). The Item Analysis of Pediatric Sleep Questionnaire Based on the Item Response Theory. Chin. Gen. Pract..

[13] Zhang B.Y., Feng D.N., Lin H.J., Tu G.T. (2024). Application and valence analysis of the simplified Chinese version of the PSQ questionnaire in the diagnosis of sleep apnea syndrome in children. J. Hebei Med. Univ..

[14] Li X.D., Tai J., Xu Z.F., Peng X.X., Feng G.S., Zhang Y.M., Zhang J., Guo Y.L., Wu Y.X., Shi J. (2016). The validity and reliability of simplified Chinese version of the pediatric sleep questionnaire for screening children with obstructive sleep apnea syndrome in Beijing. Chin. J. Otorhinolaryngol. Head Neck Surg..

[15] Saraya T., Watanabe T., Tsukahara Y., Ohkuma K., Ishii H., Kimura H., Yan K., Goto H., Takizawa H. (2017). The Correlation between Chest X-ray Scores and the Clinical Findings in Children and Adults with *Mycoplasma pneumoniae* Pneumonia. Intern. Med..

[16] The Subspecialty Group of Respiratory, the Society of Pediatrics, Chinese Medical Association, the Editorial Board, Chinese Journal of Pediatrics, China Medicine Education Association Committee on Pediatrics (2024). Guidelines for the management of community-acquired pneumonia in children (2024 revision). Chin. J. Pediatr..

[17] Lee E., Lee Y.Y. (2021). Predictive Factors of the Responses to Treatment of *Mycoplasma pneumoniae* Pneumonia. J. Clin. Med..

[18] Wu B., Li R., Hao J., Qi Y., Liu B., Wei H., Li Z., Zhang Y., Liu Y. (2024). CT semi-quantitative score used as risk factor for hyponatremia in patients with COVID-19: A cross-sectional study. Front. Endocrinol..

[19] Qiu W., Ding J., Zhang H., Huang S., Huang Z., Lin M., Zhang Y., Chen Z. (2024). *Mycoplasma pneumoniae* detections in children with lower respiratory infection before and during the COVID-19 pandemic: A large sample study in China from 2019 to 2022. BMC Infect. Dis..

[20] Diaz M.H., Hersh A.L., Olson J., Shah S.S., Hall M., Edens C. (2025). *Mycoplasma pneumoniae* Infections in Hospitalized Children—United States, 2018–2024. MMWR Morb. Mortal. Wkly. Rep..

[21] Xu Y., Yang C., Sun P., Zeng F., Wang Q., Wu J., Fang C., Zhang C., Wang J., Gu Y. (2024). Epidemic features and megagenomic analysis of childhood *Mycoplasma pneumoniae* post COVID-19 pandemic: A 6-year study in southern China. Emerg. Microbes Infect..

[22] Zheng Y., Liu X.F., Liu Z.Y. (2020). Significance analysis of MP-DNA load and drug resistance in diagnosis and treatment of children with refractory mycoplasma pneumonia in children. J. Clin. Pulm. Med..

[23] Yan C., Xue G., Zhao H., Feng Y., Li S., Cui J., Ni S., Sun H. (2019). Molecular and clinical characteristics of severe *Mycoplasma pneumoniae* pneumonia in children. Pediatr. Pulmonol..

[24] Xu N., Fan L., Li L., Guo Y. (2024). Exploring the pathogenicity of *Mycoplasma pneumoniae*: Focus on community-acquired respiratory distress syndrome toxins. Microb. Pathog..

[25] Wang Y., Yao H.Y. (2023). Research progress on immune mechanisms and predictive indicators of severe *Mycoplasma pneumoniae* pneumonia in children. Zhejiang J. Integr. Tradit. Chin. West. Med..

[26] Yang X., Li Y., Ma Y.L. (2021). Clinical and immunological characteristics of Mycoplasma pneumonia in children with atopic and non atopic constitution. Int. J. Respir..

[27] Fan F., Tang L.P., Niu H.H., Li Y.X. (2021). Analysis of the influence factors of repeated respiratory tract infection in children. Lab. Med. Clin..

[28] Epstein S., Jun D., Deng J.C., Zeidler M. (2024). Effects of Obstructive Sleep Apnea on Airway Immunity and Susceptibility to Respiratory Infections. Sleep Med. Clin..

[29] Keto J., Feuth T., Linna M., Saaresranta T. (2023). Lower respiratory tract infections among newly diagnosed sleep apnea patients. BMC Pulm. Med..

[30] Li X., Liu X., Meng Q., Wu X., Bing X., Guo N., Zhao X., Hou X., Wang B., Xia M. (2022). Circadian clock disruptions link oxidative stress and systemic inflammation to metabolic syndrome in obstructive sleep apnea patients. Front. Physiol..

[31] Boira I., Chiner E. (2025). Sleep and Respiratory Infections. Semin. Respir. Crit. Care Med..

[32] Nemet M., Vukoja M. (2024). Obstructive Sleep Apnea and Acute Lower Respiratory Tract Infections: A Narrative Literature Review. Antibiotics.

[33] Qin Y., Yang Y., Li J., Guan J. (2025). The impact of atopy on the clinical characteristics of *mycoplasma pneumoniae* pneumonia in pediatric patients. Sci. Rep..

[34] Bian C., Li S., Huo S., Yang B., Wang P., Li W., Ding S. (2023). Association of atopy with disease severity in children with *Mycoplasma pneumoniae* pneumonia. Front. Pediatr..

[35] Wang Z., Sun J., Liu Y., Wang Y. (2019). Impact of atopy on the severity and extrapulmonary manifestations of childhood *Mycoplasma pneumoniae* pneumonia. J. Clin. Lab. Anal..

[36] Hu J., Ye Y., Chen X., Xiong L., Xie W., Liu P. (2022). Insight into the Pathogenic Mechanism of *Mycoplasma pneumoniae*. Curr. Microbiol..

[37] Liu Y., Cui Y.X. (2023). Research progress on Th1/Th2 cytokine profiles and related immune mechanisms in children with *Mycoplasma pneumoniae* pneumonia. Guizhou Med. J..

[38] Yao H.S., Liu L.Y., Yi L.L., Han L.N., Zhou Q.L., Li M., Han X.H. (2020). Clinical characteristics of children with atopic *mycoplasma pneumoniae* pneumonia. Int. J. Pediatr..

[39] Zhao X., Lv J., Wu M., Wu Q. (2024). Clinical characteristics and risk factors for *Mycoplasma pneumoniae* pneumonia in children. Front. Pediatr..

[40] He W., Yin J., Wan Y. (2022). Correlations of Different Serological Parameters with the Severity and Prognosis of Pneumonia in Children Infected with *Mycoplasma pneumoniae*. Clin. Lab..

[41] Zheng Y., Hua L., Zhao Q., Li M., Huang M., Zhou Y., Wang Y., Chen Z., Zhang Y. (2021). The Level of D-Dimer Is Positively Correlated with the Severity of *Mycoplasma pneumoniae* Pneumonia in Children. Front. Cell Infect. Microbiol..

[42] Niu Y.H., Sun C., Wang C., Jiang K., Dong X.Y. (2023). Analysis of risk factors in children with severe *Mycoplasma pneumoniae* pneumonia. Shanghai Med. J..

[43] Li L., Guo R., Zou Y., Wang X., Wang Y., Zhang S., Wang H., Jin X., Zhang N. (2024). Construction and Validation of a Nomogram Model to Predict the Severity of *Mycoplasma pneumoniae* Pneumonia in Children. J. Inflamm. Res..

[44] Wang S., Jiang Z., Li X., Sun C., Zhang Y., Xiao Z. (2023). Diagnostic value of serum LDH in children with refractory *Mycoplasma pneumoniae* pneumoniae: A systematic review and meta-analysis. Front. Pediatr..

[45] Xue G., Zhao H., Yan C., Li S., Cui J., Feng Y., Xie X., Yuan J. (2021). Evaluation of the CARDS toxin and its fragment for the serodiagnosis of *Mycoplasma pneumoniae* infections. Eur. J. Clin. Microbiol. Infect. Dis..

[46] Wang T., Sun H., Lu Z., Jiang W., Dai G., Huang L., Wang M., Zhu C., Wang Y., Hao C. (2022). The CARDS toxin of *Mycoplasma pneumoniae* induces a positive feedback loop of type 1 immune response. Front. Immunol..

[47] Jia R., Guo H., Lu A., Zhang C., Qi Y., Wang D., He W., Wang Q., Cheng Z., Gao Y. (2025). Immunological landscape of children with *Mycoplasma pneumoniae* pneumonia in the post-COVID-19 era reveals distinctive severity indicators. Respir. Res..

[48] Bao Y., Shu C., Liu Y., Cao A., Zhang R., Zhao D., Liu F. (2026). Peripheral blood inflammatory cytokines linked to clinical outcomes in *Mycoplasma pneumoniae* pneumonia. Microbiol. Spectr..

[49] Esposito S., Droghetti R., Bosis S., Claut L., Marchisio P., Principi N. (2002). Cytokine secretion in children with acute *Mycoplasma pneumoniae* infection and wheeze. Pediatr. Pulmonol..

[50] Pevernagie D.A., Gnidovec-Strazisar B., Grote L., Heinzer R., McNicholas W.T., Penzel T., Randerath W., Schiza S., Verbraecken J., Arnardottir E.S. (2020). On the rise and fall of the apnea-hypopnea index: A historical review and critical appraisal. J. Sleep Res..

[51] Lévy P., Tamisier R., Pépin J.L. (2020). Assessment of sleep-disordered-breathing: Quest for a metric or search for meaning?. J. Sleep Res..

[52] Atceken Z., Celik Y., Atasoy C., Peker Y. (2024). Association of High-Risk Obstructive Sleep Apnea with Artificial Intelligence-Guided, CT-Based Severity Scores in Patients with COVID-19 Pneumonia. J. Clin. Med..

[53] Wang G.T., Zhao L.L., Liu H.Q., Zhang X. (2021). The Interaction between Neo-Crown Pneumonia and Low Ventilation Syndrome of Obstructive Sleep Apnea Was Analyzed. Adv. Clin. Med..

[54] Lv R., Liu X., Zhang Y., Dong N., Wang X., He Y., Yue H., Yin Q. (2023). Pathophysiological mechanisms and therapeutic approaches in obstructive sleep apnea syndrome. Signal Transduct. Target. Ther..

[55] Zeng X.Z., Gao W., Cui X.G. (2007). Effect of hypercapnia on neutrophil function in acute lung injury. Int. J. Anesthesiol. Resusc..

[56] Rai S., Engelberts D., Laffey J.G., Frevert C., Kajikawa O., Martin T.R., Post M., Kavanagh B.P. (2004). Therapeutic hypercapnia is not protective in the in vivo surfactant-depleted rabbit lung. Pediatr. Res..

[57] Tsioumpekou M., Krijgsman D., Leusen J.H.W., Olofsen P.A. (2023). The Role of Cytokines in Neutrophil Development, Tissue Homing, Function and Plasticity in Health and Disease. Cells.

[58] Li Y.R., Wang H.J., Han D.M. (2022). The source role in children with sleep-disordered breathing. J. Cap. Med. Univ..

[59] Mettelman R.C., Allen E.K., Thomas P.G. (2022). Mucosal immune responses to infection and vaccination in the respiratory tract. Immunity.

[60] Ito S., Aoyagi Y., Hirata M., Ohashi M., Kagaya H., Inada H., Kimura A., Shikano K., Kaneko M., Okano T. (2024). Deglutition dynamics of patients with obstructive sleep apnea. Fujita Med. J..

[61] Tamin S., Siregar D., Hutauruk S.M., Restuti R.D., Rachmawati E.Z.K., Bardosono S. (2022). Association between laryngopharyngeal reflux and obstructive sleep apnea in adults. PeerJ.

[62] Barbera L., Pasta A., Calabrese F., Zentilin P., Fragale M., Barbieri M., Peretti G., Savarino E.V., Giannini E.G., Marabotto E. (2024). Evaluation of the pathophysiological association between the GERD and OSAS: Esophageal pH-monitoring results beyond Lyon criteria. J. Gastroenterol. Hepatol..

